# Cannula complications using elastomeric infusers in Hospital in the Home

**DOI:** 10.1093/jacamr/dlaa033

**Published:** 2020-07-07

**Authors:** Damian Ryan, Jennifer Miller, Joanne Campbell

**Affiliations:** 1 Hospital in the Home, Wollongong Hospital, Illawarra Shoalhaven Local Hospital District, Wollongong, NSW, Australia; 2 Ambulatory Care Centre, Wollongong Hospital, Illawarra Shoalhaven Local Hospital District, Wollongong, NSW, Australia

## Abstract

**Objectives:**

Comparison of the short peripheral cannula (SPC) complication rate of patients with cellulitis receiving IV cefazolin via an elastomeric infuser with those receiving twice-daily bolus treatment (control group) in the Hospital in the Home service.

**Methods:**

A randomized controlled study using elastomeric infuser versus bolus delivery of IV cefazolin via an SPC of patients referred to the Hospital in the Home service in the Northern Illawarra for treatment of cellulitis. A total of 104 patients were enrolled during the time period of May 2018 to January 2019. Primary outcome measures were SPC complications including phlebitis with a secondary outcome of patient satisfaction.

**Results:**

A total of 104 patients enrolled. After randomization there were 60 in the infuser group and 44 in the bolus group. Patient characteristics of age, gender, weight and mobility were similar for the two groups. There was no statistically significant difference between the groups for the endpoint of cannula complication rates. Patient satisfaction scores showed patient acceptance of both forms of treatment.

**Conclusions:**

This study suggests that using elastomeric infusers to deliver cefazolin via a short peripheral IV catheter has similar complication rates to traditional bolus delivery. Patients surveyed showed high levels of satisfaction with both forms of antibiotic delivery.

## Introduction

The Illawarra Shoalhaven Local Health District (ISLHD) is a metropolitan health service on the east coast of New South Wales (NSW), Australia with a population of approximately 400 000 persons.^1^ It covers the local government areas of Wollongong, Shellharbour, Kiama and Shoalhaven. This catchment area is approximately 250 km long. The northern region covers the local government areas of Wollongong, Shellharbour and Kiama and is referred to as the Northern Illawarra (NI) with a population of over  260 000.^2^ This research project was conducted at the Hospital in the Home (HITH) service based at Wollongong Hospital, which covers the local government areas of Wollongong, Shellharbour and Kiama, referred to as the Northern Illawarra (NI).

HITH services manage outpatient parenteral antimicrobial therapy (OPAT). Home-based IV antimicrobial treatment is a safe and cost-effective method of treating patients,^3^ can have just as good, if not better, outcomes for patients than hospital admission^4^ and is highly acceptable to patients.^5^ Patients with HITH-style care have the advantage of avoiding the complications associated with being in hospital^6^ and the benefits of being managed in their own home environment, which include fewer medication errors, less confusion, lower infection rates and easier carer access.^7^ Patients and carers report high levels of satisfaction with HITH services.^8^^,^^9^ HITH provides benefits for the hospital system, particularly in the areas of access block and the cost of in-hospital care.^10^^,^^11^

For soft tissue infections such as cellulitis, HITH is a safe and effective way of managing these in the community.^3^^,^^12^ In NSW, cellulitis is the leading diagnostic-related group (DRG) for HITH services.^1^ Likewise, in the NI, cellulitis is the most common DRG managed by HITH and of the over 1200 admissions in 2018,^13^ around 50% were for soft tissue infections and the majority of these were cellulitis.^13^

Treatment of cellulitis in HITH involves a multidisciplinary approach but the principle management is the use of IV antibiotics. Traditionally this is done by bolus injections of antibiotics using a short peripheral cannula (SPC). The advantages of bolus treatments are that they allow a quick response, achieve high blood concentrations and use a small volume of fluid.^14^ There are limitations on the frequency of dosing of antibiotics in HITH due to service logistics, particularly for services that are required to cover large geographical areas. For this reason, HITH NI sets a maximum dosing frequency of twice a day. If antibiotics are required to be given more frequently than this, the only alternative is a continuous infusion via an elastomeric infuser. Infusers have the advantage for the service of only requiring once-daily visits. When connected to a peripherally inserted central catheter (PICC), the patient can retain their mobility and independence at home.^15^

Current HITH NI guidelines state that elastomeric infusers must be connected to a central line such as a PICC. PICCs are invasive, require specially trained technicians to insert and need significantly more maintenance than an SPC. Despite this, they provide reliable long-term venous access.^16^ SPCs have the benefit of being readily available, requiring less training and equipment for insertion and requiring less maintenance.^17^ An SPC costs approximately one eighth of the cost of a PICC line.^18^

Using an elastomeric infuser via an SPC in HITH gives the advantage of quick, easy and cheaper venous access coupled with a once-daily home visit. The concern of using elastomeric infusers via an SPC is dislodgement and extravasation of the antibiotic causing tissue damage due to pH and/or irritant properties^18^ and phlebitis.

Our study compared the cannula complication rates of the two methods of antibiotic delivery to document the standard therapy rate and compare and contrast that with the infuser rate. Secondary outcomes were patient satisfaction.

## Methods

### Study setting and design

This study was a prospective randomized study conducted by HITH NI, a multidisciplinary service based in the Ambulatory Care Clinic in Wollongong Hospital. The patients were classified as inpatients and admitted under an HITH staff specialist. Patients were managed in the home setting, including aged care facilities, for the majority of their care and presented to the clinic for medical review or for all their treatment if there were work, health and/or safety issues. The majority of care was delivered by HITH nurses.

The research project had ethics approval granted via the University of Wollongong (UOW) and ISLHD Human Research Ethics Committee (HREC). (Ethics Number: 2016/90. AuRed Number: HREC/16/WGONG/191. Review Date 18 October 2016). The Health and Medical HREC reviewed the proposal in accordance with the National Health and Medical Research Council National Statement on Ethical Conduct in Human Research to ensure that the project was consistent with the requirements of the National Statement.^19^

Patients were assessed for the study between May 2018 and January 2019. All patients were adults and had a diagnosis of cellulitis. Patient referral sources were the emergency department of Wollongong Hospital, the emergency department of Shellharbour hospital or GPs in the NI. Participation in the trial required the patient to be diagnosed with cellulitis in the 24 h prior to referral and had to have received one bolus dose of cefazolin 2 g. This avoided any delay in treatment for patients referred from the emergency department outside HITH operational hours. Once a patient was identified to be suitable for the trial, the trial information was provided and consent obtained. They were then randomized into the two groups: one receiving cefazolin 2 g twice daily as a bolus injection, the other receiving cefazolin 6 g over 24 h via an elastomeric infuser. Simple randomization was done by a coin toss by a clerical member not involved in the study. Patient variables and demographics recorded included age, sex, weight, mobility score, diagnosis, cannula size, cannula site, referral source, number of treatment days, number of SPCs and reason for peripheral cannula change. All cannulas used were the same brand and material.

The SPCs were assessed each day for complications by the HITH nurses, daily in the elastomeric group and twice daily in the bolus group. The cannula complications were divided into four groups: occlusion, accidental removal, leaking and phlebitis. Diagnosis of phlebitis was based on clinical signs of pain, erythema or swelling. A visual phlebitis scale was provided for each nurse to standardize reporting. Occlusion was defined as a non-patent cannula with no clinical signs of phlebitis and leaking was defined as a visible fluid around the cannula site, again without the clinical signs of phlebitis. Any of these findings resulted in immediate removal of the cannula. SPC outcomes were documented as ‘complete’ if they were removed as per protocol or at the finish of treatment with no complications.

### Participants

All patients referred to HITH with a diagnosis of cellulitis were identified by the HITH Care Navigator (a registered nurse) and screened as appropriate for inclusion in the study. Patients resided in the catchment area of HITH NI. Patients were adults (minimum age 18 years) and had a diagnosis of cellulitis made by a medical practitioner or emergency nurse practitioner.

Inclusion criteria were uncomplicated cellulitis, the patient having received one dose of cefazolin, the patient having consented to being in the trial and passed HITH NI standard criteria (must be contactable by phone, must be able to care for self or have a carer and must reside in the NI). Exclusion criteria were complicated cellulitis, patients who had received more than one dose of IV antimicrobial before referral, non-English speaking, patient declined invitation to be part of the trial, allergy to cephalosporins, any work, health and/or safety issues or failed HITH NI standard criteria.

The study enrolment target was 100 patients to be consistent with similar research.^20^^,^^21^

### Data collection

Records were kept in a paper folder and an Excel spreadsheet. The paper record documented their medical record number, gave each patient a study number and was stored in a locked office in the HITH department. The Excel spreadsheet was stored on an ISLHD server and the patients were identified by their study number and date of birth (DOB) only. Thus only the investigators could link the patients with their medical record number, DOB and study number. Deidentified data were transferred to Redcap (research database capture tool) and downloaded to an Excel spreadsheet for analysis by the research clinician who was employed by the area health service in the research department known as ‘Research Central’. Additional data were sourced from the HITH patient registry and the NSW electronic medical record (eMR).

Data collected for the trial included medical record number, age, sex, diagnosis, mobility score and weight. Treatment parameters included diagnosis, cannula size, cannula site, cannula time *in situ*, cannula complications (if any) and date of insertion and removal. A patient satisfaction survey and a staff satisfaction survey were conducted as an additional part of the trial to look at acceptability of the intervention (available as Supplementary data at *JAC-AMR* Online).

### Outcomes

The primary outcome was the number of SPC complications, which were defined as either mechanical (accidental removal, leakage or blocked) or clinical (phlebitis or thrombophlebitis) and resulted in an intervention. Phlebitis was measured using the phlebitis scale.^22^ The secondary outcome was patient acceptability.

### Statistical analysis

All statistics were calculated via Stata/IC v14.2. Descriptive statistics, including the mean and standard deviation for continuous variables and frequencies and percentages for categorical variables, were captured to compare the numbers between the bolus and infuser groups. The complication rates for both the bolus and infuser groups were calculated as the number of people who experienced a complication divided by the total number of people in each group. Both a *χ*^2^ test and a two-proportion *z*-test were then used to compare the complication rates.

## Results

A total of 104 patients participated in the study, with a total of 141 cannulations. Descriptive characteristics of participants in the infuser and bolus groups are outlined in Table 1. No statistically significant differences in patient characteristics between the two groups (weight, age, gender and mobility) were shown by *χ*^2^ tests.

**Table 1.** Patient characteristics

Patient characteristics, *n* (%) or mean (SD)	Bolus	Infuser
Age (years)	52.9 (21.22)	54.6 (17.66)
Gender		
male	27 (61.4)	37 (61.7)
female	17 (38.6)	23 (38.3)
Weight (kg)	89.6 (22.75)	95.77 (22.05)
Mobility score		
1—independent	41 (93.2)	54 (90.0)
2—assist × 1	3 (6.8)	6 (10.0)
3—bed bound	—	—

In the study, all patients were cannulated once, 31 had a second cannula and 3 had a third cannula. In the infuser group, 37 patients had one SPC, 20 had two SPCs and 3 had three SPCs, whereas in the control (bolus) group, 33 had one cannula and 11 required two (Table 2). There was no difference between the two groups using *P* value.

**Table 2.** SPC information

SPC information, *n* (%)	Bolus	Infuser
Number of cannulas		
1	33 (75.0)	37 (61.7)
2	11 (25.0)	20 (33.3)
3	—	3 (5.0)
Side		
left	30 (54.5)	47 (54.7)
right	25 (45.5)	39 (45.4)
Size (g)		
18	4 (7.3)	10 (11.6)
20	26 (47.3)	32 (37.2)
22	24 (43.6)	44 (51.2)
24	1 (1.8)	—
Site		
hand	11 (20.0)	17 (19.8)
arm/forearm	28 (50.9)	43 (50.0)
cubital fossa	10 (18.2)	18 (20.9)
wrist	6 (10.9)	8 (9.3)

### SPC size

In this study all the cannulas were made of the same material and were the same brand; only the gauge varied. Of the 141 SPCs used in the study, 14 were 18 g, 58 were 20 g, 68 were 22 g and 1 was 24 g (Table 2). Of the first cannulations, 20 g was the commonest size, with 48% being in the infuser group and 52% being in the control group. The second cannulations used predominantly 22 g, 78% for the infuser group versus 63% for the control group, and the third cannulations all used 22 g and were all from the infuser group.

### SPC site

The cannula sites were statistically similar between the two groups. The infuser group had 54.7% of SPCs in the left side and 50% in the forearm. The control group had 54.5% in the left side and 50.9% in the forearm. SPC site information is shown in Table 2.

### SPC placement time

Total number of treatment days was 551 for both groups; 342 days for the infuser group and 209 days for the control group. The infuser group averaged 5.7 days per participant and an average SPC dwell time of 3.98 days. The control group averaged 4.75 days per participant and had an average SPC dwell time of 3.8 days per cannula. Cannula dwell times are shown in Table 3.

**Table 3.** SPC dwell times

Dwell time (days)	Bolus	Infuser	Total
Cannula 1	167	250	417
Cannula 2	42	82	124
Cannula 3	—	10	10
Total	209	342	551

### SPC complications

The overall SPC complication rate was 25.5% (36/141). The infuser group had a complication rate for the first SPC of 30% (18/60), a complication rate for the second SPC of 17.4% (4/23) and for the third SPC of 0% (0/3), giving an overall complication rate of 25.6%. The control group had a complication rate for the first SPC of 22.7% (10/44) and for the second SPC of 36.4% (4/11), giving an overall complication rate of 25.5%.

The commonest complication in both groups was ‘occlusion’, affecting 36% (8/22) of the infuser group and 35.7% (5/14) of the control group (Table 4).

**Table 4.** SPC complication results

SPC complication information (%)	Bolus	Infuser	*P* value
Number of complications			0.897
0	12 (27.3)	40 (66.7)	
1	1 (2.3)	18 (30.0)	
2		2 (3.3)	
Was there a complication?			0.841
no	41 (74.5)	64 (74.42)	
yes	14 (25.5)	22 (25.58)	
Complication type			0.987
accidental removal	2 (14.3)	4 (18.2)	
leaking	3 (21.4)	7 (31.8)	
occlusion	5 (35.7)	8 (36.4)	
phlebitis	4 (28.6)	3 (13.6)	

The first SPCs were inserted by emergency department staff or HITH NI staff. The second and third SPCs were all inserted by HITH NI staff.

## Discussion

This study is novel research, as it documented the complication rate of SPCs with standard bolus treatment and compared that with elastomeric antibiotic delivery, whereas previous similar studies documented the elastomeric SPC complication rate only. The study’s vascular access complication rate was 25.6% in the infuser group and 25.5% in the bolus group, a similar rate to that found in other research.^20^^,^^21^ The phlebitis rate was 5% in the infuser group and 9% in the bolus group. Documented rates of phlebitis can vary from 2% to 80%.^23^

Our results were comparable with other similar research. King *et al*.^20^ provided evidence that an elastomeric infuser can be used safely with an SPC, with an overall cannula complication rate of 6%. Ryan-Agnew^21^ looked at the incidence of phlebitis in SPCs using elastomeric infusers. In their study of 82 patients, it showed a phlebitis rate with cefazolin of 8% versus 5% via a PICC line. Poole *et al*.^17^ showed no significant difference in the development of phlebitis between IV bolus and minibag delivery of antibiotics.

There are limitations to our study. The sample size in the study was small. Future studies should consider using a larger sample size. Consideration was given to extending the number of patients; however, the study ethics application was for 100 patients. Also, despite randomization there were more patients in the infuser group, which may have introduced bias in the outcomes. We could find no reason why the groups varied in their numbers.

Another factor that could have created bias was the technique of insertion of the first cannula^24^ as this was done outside the study. The majority of the first cannulations were done by emergency department staff whereas the second and third cannulations were done by the HITH medical officers or registered nurses. Research shows a difference in complication rates of SPC depending on the skills of the staff inserting them,^25^^,^^26^ with emergency department nurses having a greater rate of phlebitis.^27^ Our study results showed that all second and third cannulas were done by HITH staff who primarily used 22 g SPCs. In this group the SPC complication rate was higher than the first group, a group where the commonest gauge was 20 g and a significant proportion were inserted by emergency department staff. SPCs in the study were secured, dressed and the dressing changed in the same manner using a standardized hospital policy. Security of the SPC in the infuser group could have been influenced by being connected continuously to an IV line.

Studies have shown that patients may have a predisposition to phlebitis^28^ and this could affect outcomes. In one study, patients were 5.1 times more likely to have phlebitis a second time if they had phlebitis with their first cannula.^28^ Research also shows that the rate of phlebitis increases among patients with two or more SPCs.^2^ In our study, of the 22 who had complications with the first cannula, 8 of them had a complication with the second.

Research has shown that there are many alternative risk factors implicated in developing infusion-related phlebitis.^14^ The nature of this study and similarity of the patient demographics removed some of these biases, which include female gender, age >60 years, SPC size, SPC material, SPC duration, infusate characteristics and cannula dressing changes. However, the study didn’t allow for the bias of quality of veins, underlying medical conditions, experience of the person inserting the SPC nor the department by whom the SPC was inserted.^14^

Important factors considered when measuring phlebitis rates are that there are three possible causes: chemical, due to the pH and/or the osmolality of the infusate; mechanical, due to insertion and stabilization; and bacterial infection.^28^ The pH of antibiotics affects the rate of phlebitis^17^ but the pH of the antibiotic was the same for both groups. Phlebitis is associated with infusion of hyperosmolar fluids (greater than 600 mOsm).^28^ The different modalities of antibiotics in the study meant that there were different osmolality values for each solution. The infuser delivered 6000 mg in 240 mL, which is 25 mg/mL, while the bolus delivered 2000 mg in 20 mL, which is 100 mg/mL, four times the concentration of the infuser.

Clinically it may be difficult to differentiate the cause of SPC malfunction and the stigmata of phlebitis and acknowledge that not all phlebitis may display clinical signs.^28^ HITH nurses were educated about phlebitis and cannula complications but there may have been variation within the skill sets of the nurses in categorizing SPC complications and what may have been reported as occlusion or leaking may in fact have had associated phlebitis.^14^

Further bias was noted in that the bolus group was assessed twice a day and the infuser group was only assessed once a day, resulting in the SPCs in the bolus group being scrutinized twice as often.

### Patient satisfaction

A patient satisfaction survey was given to participants (see Supplementary data). The response rate was 67% overall, with 49 of the infuser group (82%) and 21 of the bolus group (48%) responding. A summary of the results is in Table 5. Patients were asked to evaluate, on a scale of 0 to 10 (0 = unhappy, 5 = neutral, 10 = very happy), how well the treatment was explained to them (Question 1) and how happy they were with managing their treatment (Question 3). Two respondents had used an elastomeric infuser before (Question 2). A total of 87.5% of respondents felt safe having their treatment at home (Question 4). Question 4 was the only question with a significant difference between the groups; however, eight participants in the bolus group wrote ‘N/A’ and three didn’t respond to the question. Eighty-five percent of patients did not need to call the service with concerns regarding the treatment (Question 5) and 98.5% felt supported with their treatment (Question 6). These results are similar to other research showing both modalities are acceptable to patients. Poole *et al*.^17^ used a scale of 1–4 and their results were IV push 99% (3 or 4) and elastomeric infuser 96% (3 or 4).

**Table 5.** Patient satisfaction survey results

Patient satisfaction survey results	Elastomeric infuser (*n *=* *49)	Bolus (*n *=* *21)
Q1: Nurse explanation (0–10, average)	9.75	9.89
Q2: Used elastomeric infuser before (yes)	1	1
Q3: Happy with understanding (0–10, average)	9.76	9.56
Q4: Safe having antibiotics (yes, %)^a^	94	48
Q5: Need to call HITH service (yes, %)	12	9.5
Q6: Felt supported with treatment (yes, %)	92	90
Q7: Would you recommend treatment (yes, %)	96	95

^a^Question 4 introduced bias in the results as it was directed at the infuser group and excluded the bolus group.

The design of the survey introduced some bias as the wording of the questions was targeted towards the infuser group. On reflection, the wording of Question 4 should have been altered to make it inclusive of both groups.

### Conclusions

Similar SPC complication rates were found for elastomeric infuser and bolus antibiotic delivery for patients in this study. From this we can conclude that elastomeric infusers connected to SPCs have comparable complication rates to using the traditional bolus delivery of antibiotics. The advantages for an HITH service is allowing more options for patient treatment with the cost benefits, ease of access of using an SPC and once-daily visiting. This will change practice in our HITH service.

## Supplementary Material

dlaa033_Supplementary_Data

## Supplementary data

The Reviewer report and Survey are available as Supplementary data at *JAC-AMR* Online.
