# Supplementary material for: Cannula complications using elastomeric infusers in Hospital in the Home
Source: JAC Antimicrob Resist. 2020 Jul 7;2(3):dlaa033. doi: 10.1093/jacamr/dlaa033 (PMC8210130; doi:10.1093/jacamr/dlaa033)
Supplement: dlaa033_Supplementary_Data [file dlaa033_supplementary_data.zip › Supplementary_Data.doc]

**Participant Survey**

**HIPE Study**

**“Hospital in the Home (HITH) Interventional Trial of Peripheral Line Cannulas with an Elastomeric Infuser”**

This survey is being conducted to explore your experience of having received intravenous antibiotic treatment through Hospital in the Home (HITH). This will help us improve our service. Your responses are anonymous and it will take about 10 minutes to complete this survey.

**Thank you for your time in completing the following survey.**

1. During the admission process to Hospital in the Home, how well did the HITH nurse(s) explain the functions of the elastomeric infuser device (Baxter bottle) in a way you could understand? Circle the appropriate number:

Unhappy	Feel neutral	Very Happy

2. Have you ever used an elastomeric infuser device (Baxter bottle) before this treatment?

Yes	No

3. How happy were you with your understanding of how to manage your elastomeric infuser device? Circle the appropriate number:

Unhappy	Feel neutral	Very Happy

4. Did you feel safe having your antibiotics administered at home with the elastomeric infuser device?

Yes	No

5. Did you ever need to call the HITH service with a concern relating to your elastomeric infuser device or cannula?

Yes	No

6. Did you feel the HITH team supported you at home with this type of treatment?

Yes	No

7. Would you recommend this type of treatment with HITH to your family and friends?

Yes	No

## Would you like to provide any feedback or suggestions relating to your experience?

Thank you for your participation
